# New-onset dyslipidemia in adult cancer survivors from medically underserved areas: a 10-year retrospective cohort study

**DOI:** 10.1186/s12885-023-11384-2

**Published:** 2023-09-26

**Authors:** Yun Hwa Jung, IL Yun, Eun-Cheol Park, Sung-In Jang

**Affiliations:** 1 Department of Public Health, Graduate School, Yonsei University, Seoul, Republic of Korea; 2 Institute of Health Services Research, Yonsei University, Seoul, Republic of Korea; 3 Department of Preventive Medicine &, Institute of Health Services Research, Yonsei University College of Medicine, 50 Yonsei-Ro, Seodaemun-Gu, Seoul, 03722 Republic of Korea

**Keywords:** Medically underserved area, Health disparities, Dyslipidemia diagnosis, Cancer survivors

## Abstract

**Background:**

Cancer survival rates are increasing; however, studies on dyslipidemia as a comorbidity of cancer are limited. For efficient management of the disease burden, this study aimed to understand new-onset dyslipidemia in medically underserved areas (MUA) among cancer survivors > 19 years.

**Methods:**

This study used 11-year (2009–2019) data from the Korean National Health Insurance Service sample cohort. Cancer survivors for five years or more (diagnosed with ICD-10 codes ‘C00-C97’) > 19 years were matched for sex, age, cancer type, and survival years using a 1:1 ratio with propensity scores. New-onset dyslipidemia outpatients based on MUA were analyzed using the Cox proportional hazards model.

**Results:**

Of the 5,736 cancer survivors included in the study, the number of new-onset dyslipidemia patients was 855 in MUA and 781 in non-MUA. Cancer survivors for five years or more from MUA had a 1.22-fold higher risk of onset of dyslipidemia (95% CI = 1.10–1.34) than patients from non-MUA. The prominent factors for the risk of dyslipidemia in MUA include women, age ≥ 80 years, high income, disability, complications, and fifth-year cancer survivors.

**Conclusions:**

Cancer survivors for five years or more from MUA had a higher risk of new-onset dyslipidemia than those from non-MUA. Thus, cancer survivors for five years or more living in MUA require healthcare to prevent and alleviate dyslipidemia.

## Background

The age-standardized incidence of all cancers per 100,000 people in Korea was 221.7 in 1999 and 295.8 in 2019 [1]. When Koreans had a life expectancy of 83 years, the cumulative cancer risk to life expectancy was 37.9% [2]. However, cancer relative survival rates are increasing. For all cancers in Korea, the 5-year relative survival rates for patients were as follows: between 2001 and 2005, 54.1%; between 2006 and 2010, 65.5%; and since 2011, 70.7% [3]. The 5-year relative survival rates for all cancers were 66.1% (prevalence 2004–2010) in the United States [4], 63% (prevalence 2006–2008) in Canada [5], and 58.6% (prevalence 2003–2005) in Japan [6, 7].

Healthy life years after cancer survival are as important as cancer treatment. The overall quality of life of cancer survivors is lower than that of the general population. The EuroQol-5 Dimension score for those > 19 years was 0.95 in the general population and 0.90 in cancer survivors [2]. The risk of complications and chronic diseases is high among cancer survivors. Cancer survivors with comorbidities have a higher risk of developing secondary cancer. However, previous studies on cancer survivors with dyslipidemia, a comorbidity, are limited [7–9]. Although dyslipidemia is a pervasive chronic disease, its management and attention are relatively limited. The prevalence of dyslipidemia among Korean adults was 45.6% in men and 31.3% in women [10], and there was a tendency for a positive relationship with age in adults under the age of 60 [11, 12]. Dyslipidemia is a condition in which total cholesterol, low-density lipoprotein (LDL) cholesterol, and triglycerides (TG) in the blood are increased, or high-density lipoprotein (HDL) cholesterol is decreased. The number of new dyslipidemia cases is expected to increase as it is caused by lifestyle factors such as fat-based diets, lack of exercise, genetic factors, diseases, and drugs [13]. Although dyslipidemia can be controlled with statin drugs [14], it is difficult to cure, and most cases are asymptomatic and detected by blood tests [15].

As this chronic disease can lead to life-threatening cardiovascular diseases such as atherosclerosis, myocardial infarction, and stroke, it is a chronic disease for which prevention and management are important [16]. This dyslipidemia is important in cancer survivors for several reasons. Because a history of cancer is a risk factor for deteriorating health and disability [17]. The cancer survivor population was at high risk of chronic disease, not only in middle-aged and older adults but also in adolescents and young adults [17, 18]. This high symptom burden was also present in cancer survivors [19]. Nonetheless, previous research on the healthcare of cancer survivors includes fragmentary, therefore research areas still remain. In addition, health care for chronic diseases such as hypertension, diabetes, and dyslipidemia in cancer survivors is especially meaningful for those who have been diagnosed for more than five years. This is because patients undergoing active cancer treatment can have anorexia and cachexia due to cancer, and patients with terminal cancer can stop prescriptions with palliative care [20, 21].

In addition, medical deficiencies can be another determinant of health status. According to the World Health Organization, access to and use of health care services are key determinants of health [22]. Additionally, a lack of medical infrastructure tends to increase the risk of chronic disease [23]. Therefore, identifying the dyslipidemia status of cancer survivors according to medically underserved areas (MUA) can contribute to finding ways to reduce health inequalities.

A selective method is used to prevent and manage dyslipidemia in cancer survivors. Understanding disease onset and severity in MUA will allow for the use of limited and inelastic medical resources in a cost-effective manner [24]. The determinants of MUA include local health demand (people, income, mobility and living, health determinants), supply (medical personnel and facilities, resources), accessibility (proportion accessible within a standard time), medical use (facility utilization rate, screening rate), and health outcomes (cure, death) [25]. Although the number of medical staff in Korea is small, there are several medical activities. The number of clinicians per 1,000 people is 2.5, and that of nursing staff is 7.9 (Organization for Economic Cooperation and Development [OECD] average: 3.6 doctors, 9.4 nursing staff). Nevertheless, the number of outpatient treatments per person is 17.2 yearly, the highest among OECD countries. The total number of hospital beds is 12.4 per 1,000 people, which is approximately 2.8 times the OECD average [26]. Intensive medical practice in Korea is concentrated in metropolitan areas and large cities.

The definition of medically vulnerable areas is diverse, and it is difficult to agree on an absolute standard. Therefore, this study used data from a sample cohort to compare the level of healthcare between regions. The purpose of this study was to understand the occurrence of dyslipidemia among cancer survivors for five years or more living in medically vulnerable areas.

## Methods

### Data

This study used the data of a sample cohort (2009 to 2019) from the Korea National Health Insurance Service (NHIS). In 2006, the NHIS collected data from 1 million people of the approximately 48 million Korean population. Data before 2005 were collected retrospectively, whereas data from 2006 were prospectively surveyed. Sampling was stratified by sex, age, region, health insurance coverage, and income. These data provided personal anonymized records of basic demographic and socioeconomic factors, medical treatment, health screenings, and long-term care from medical institutions. Disease records complied with the International Classification of Diseases 10th revision (ICD-10) codes. This study was approved by the Institutional Review Board (IRB) of Yonsei University Health System (IRB number: Y-2020-0031).

### Participants

The NHIS sample cohort has been documented since 2002 with 48,222,537 enrollees. Records from 2002 to 2003 were eliminated for study accuracy. Among the 38,593 cancer diagnoses, survivors of less than 5 years, those who had been diagnosed with dyslipidemia before cancer diagnosis, and individuals under the age of 19 were excluded. Then, by matching MUA, the new onset of dyslipidemia was finally analyzed for 5,736 cancer survivors for more than 5 years.

### Variables

Cancer survivors for five years or more were defined as a person who were diagnosed with ICD-10 codes ‘C00-C97’ [27] as major or minor symptoms and survived for 5 or more years. New-onset dyslipidemia was defined as the first outpatient diagnosis of ICD-10 code ‘E78’ [28] with major or minor symptoms five years after the first cancer diagnosis.

MUA is defined as a medically vulnerable area, and we calculated it as a position value for the relative comparison (PARC) index. The PARC index is a comparison method that calculates the relative position of a specific object from -1 point (inferior) to 1 point (excellent) using the median, minimum, and maximum values of the comparison group [29]. In the following formula, the PARC score was obtained from the medical demand, supply and resources, accessibility, utilization, and health outcome factors of 250 administrative districts (si, gun, and gu) in Korea.

$$\mathrm{If\ }{Value}_{region}\ge {Value}_{median,\ } {PARC}_{region}=\left(\frac{{Value}_{region}- {Value}_{median}}{{Value}_{max }- {Value}_{median}}\right),$$

$$\mathrm{Else\ if\ }{Value}_{region}<{Value}_{median,\ } {PARC}_{region}=-\left(\frac{{Value}_{region} - {Value}_{median}}{{Value}_{min }- {Value}_{median}}\right)$$

As a result, 82 regions with a PARC score of less than -0.33 were classified as relatively vulnerable [30].

Covariates were sex (men, women), age (20–29, 30–39, 40–49, 50–59, 60–69, 70–79, or 80 + years), household income (high, mid, or low), health insurance (employee health insurance, local-subscriber health insurance, or medical aid), disability (no or yes), 5-year survival after cancer onset (yes or no), Charlson comorbidity index (CCI) (0, 1, or ≥ 2), cancer type by frequency (stomach, liver, colorectal, lung or bronchus, or the other), survival years (5, 6–7, 8–9, or ≥ 10), and 5^th^ year of survival after cancer diagnosis (from 2009 to 2019).

### Statistical analyses

We matched MUA and non-MUA cancer survivors for propensity scores in a 1:1 ratio according to gender, age, cancer type, and survival years, using logistic regression analysis with greedy algorithms which of the best matches first and the next-best matches next. Descriptive statistical analyses were performed, including a chi-squared test and incidence rate with person-years, to confirm general characteristics. New-onset dyslipidemia and admission according to MUA were analyzed using the Cox proportional hazards model. Subgroup analyses were performed for sex, age, household income, disability, 5-year survival after cancer onset, CCI, and cancer type using the same analysis method. Results included adjusted hazard ratios (HRs) and 95% confidence intervals (CIs). Statistical significance was set at *P* ≤ 0.05. Statistical analyses were performed using SAS version 9.4 (SAS Institute Inc., Cary, NC, USA).

## Results

Table 1 shows the general characteristics of the study population following the new-onset of dyslipidemia outpatient. Of the 5,736 survivors, 1,636 developed new-onset dyslipidemia. For 2,868 non-MUA patients, the incidence rate per 1,000 person-years was 49.50 (incidence rate, 95% CI = 46.23–53.00). The incidence rate per 1,000 person-years for 2,868 patients from MUA was 57.53 (incidence rate, 95% CI = 53.77–61.55). At baseline, the sex ratio of the subjects was similar (men: 49.0%, women: 51.0%), the mean age was 63.3 years (standard deviation [SD]: 14.7), and the mean cancer survival was 10.7 years (SD: 3.2).

**Table 1:** General characteristics of the study population according to new onset of dyslipidemia at the baseline

**Variables**	**New onset of dyslipidemia**						
	**Subjects**		**Person-year**	**Patients**		**Incidence rate per 1,000 person-years**	**Incidence rate (95%CI)**
	**N**	**%**		**N**	**%**		
**Total**	**5,736**	**100**	30,624.38	**1,636**	**100**	53.39	(50.89–56.02)
**Medically vulnerable areas**							
Non-vulnerable	2,868	50.0	15,770.35	781	47.7	49.50	(46.23–53.00)
Vulnerable	2,868	50.0	14,854.02	855	52.3	57.53	(53.77–61.55)
**Sex**							
Men	2,812	49.0	14,901.15	733	44.8	49.17	(45.73–52.86)
Women	2,924	51.0	15,723.23	903	55.2	57.40	(53.83–61.21)
**Age (Mean: 63.3, SD: 14.7)**							
20–29	96	1.7	539.74	12	0.7	22.22	(12.72–38.82)
30–39	296	5.2	1,754.40	59	3.6	33.61	(26.09–43.30)
40–49	634	11.1	3,585.15	166	10.1	46.28	(39.90–53.68)
50–59	1,184	20.6	5,951.85	443	27.1	74.39	(67.93–81.47)
60–69	1,250	21.8	6,313.42	409	25.0	64.75	(58.88–71.20)
70–79	1,490	26.0	8,194.24	430	26.3	52.45	(47.68–57.69)
≥ 80	786	13.7	4,285.58	117	7.2	27.29	(22.74–32.74)
**Household income**							
High	2,607	45.4	14,283.93	761	46.5	53.25	(49.63–57.14)
Mid	1,849	32.2	9,970.10	526	32.2	52.73	(48.44–57.41)
Low	1,280	22.3	6,370.34	349	21.3	54.76	(49.37–60.73)
**Health Insurance**							
Employee health insurance	1,903	33.2	10,426.26	544	33.3	52.15	(48.00–56.66)
Local-subscriber health insurance	3,751	65.4	19,957.28	1,084	66.3	54.29	(51.17–57.60)
Medical aids	82	1.4	240.84	8	0.5	33.19	(16.17–68.12)
**Disability**							
No	5,007	87.3	26,831.21	1,462	89.4	54.46	(51.76–57.30)
Yes	729	12.7	3,793.17	174	10.6	45.85	(39.52–53.19)
**Charlson comorbidity index (Mean: 0.8, SD: 1.1)**					0.0		
0	2,879	50.2	15,558.97	740	45.2	47.54	(44.29–51.02)
1	1,691	29.5	9,050.18	512	31.3	56.54	(51.90–61.61)
≥ 2	1,166	20.3	6,015.23	384	23.5	63.80	(57.62–70.66)
**Cancer type**							
Stomach	764	13.3	4,000.32	165	10.1	41.23	(35.37–48.05)
Liver	506	8.8	2,935.13	148	9.0	50.40	(43.08–58.96)
Colorectal	622	10.8	3,122.27	163	10.0	52.18	(44.73–60.87)
Lung or bronchus	405	7.1	2,195.00	94	5.7	42.80	(35.07–52.24)
The other	3,439	60.0	18,371.66	1,066	65.2	57.99	(54.65–61.55)
**Survival years (Mean: 10.7, SD: 3.2)**							
5	481	8.4	1,250.08	17	1.0	13.58	(8.29–22.25)
6–7	994	17.3	3,431.49	109	6.7	31.74	(26.15–38.53)
8–9	965	16.8	4,182.18	213	13.0	50.90	(44.43–58.31)
≥ 10	3,296	57.5	21,760.63	1,297	79.3	59.58	(56.47–62.86)
**5th survival year after cancer diagnosis**							
2009	729	12.7	5,295.11	271	16.6	51.16	(45.43–57.61)
2010	736	12.8	4,956.95	283	17.3	57.07	(50.81–64.10)
2011	649	11.3	4,230.55	246	15.0	58.12	(51.43–65.69)
2012	621	10.8	3,940.16	199	12.2	50.48	(43.96–57.98)
2013	540	9.4	3,269.45	180	11.0	55.03	(47.72–63.47)
2014	533	9.3	3,089.27	155	9.5	50.15	(43.01–58.48)
2015	406	7.1	1,626.87	110	6.7	67.57	(56.23–81.19)
2016	451	7.9	1,695.27	85	5.2	50.10	(40.45–62.06)
2017	376	6.6	1,081.66	55	3.4	50.80	(38.83–66.46)
2018	384	6.7	944.96	36	2.2	38.05	(27.43–52.79)
2019	311	5.4	494.14	16	0.3	32.32	(19.13–54.60)

**Fig. 1:**
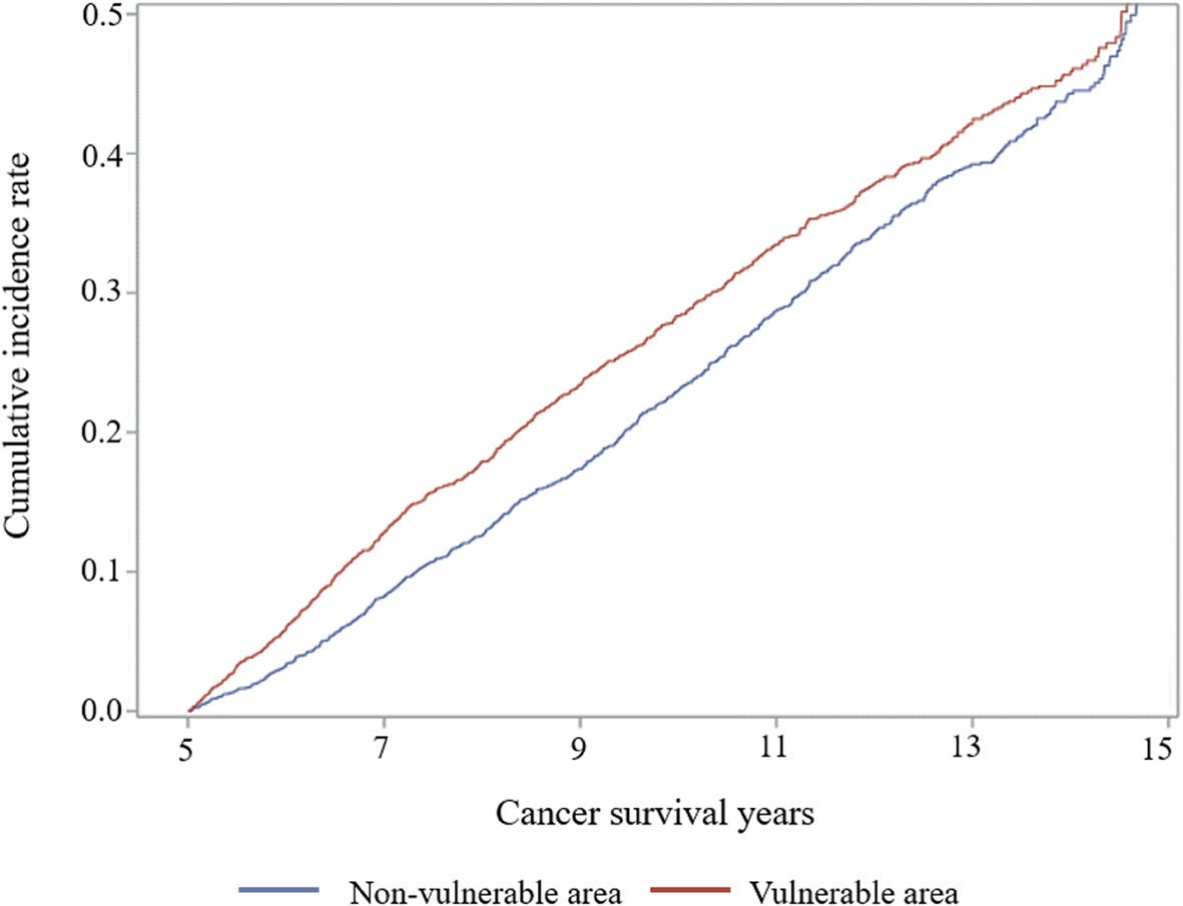
Cumulative incidence rate of new-onset risk of dyslipidemia

Table 2 shows the results of the Cox proportional hazards regression analysis of the dyslipidemia risk. Compared with non-MUA patients, MUA patients had a 1.22 times (95% CI = 1.10–1.34) higher risk of new-onset dyslipidemia. Figure 1 shows the cumulative incidence rate of new-onset dyslipidemia.

**Table 2:** Cox proportional hazards regression analysis of dyslipidemia risk

**Variables**	**Dyslipidemia**	
	**Risk of new onset**	
	**Adjusted HR**	**95% CI**
**Medically vulnerable areas**		
Non-vulnerable	1.00	
Vulnerable	1.22	(1.10–1.34)
**Sex**		
Men	1.00	
Women	1.09	(0.99–1.21)
**Age**		
20–29	1.00	
30–39	1.49	(0.80–2.78)
40–49	2.15	(1.19–3.87)
50–59	3.53	(1.98–6.27)
60–69	3.02	(1.70–5.39)
70–79	2.50	(1.40–4.46)
≥ 80	1.57	(0.86–2.86)
**Household income**		
High	1.00	
Mid	0.97	(0.86–1.08)
Low	1.04	(0.91–1.18)
**Health Insurance**		
Employee health insurance	1.00	
Local-subscriber health insurance	1.06	(0.95–1.17)
Medical aids	0.68	(0.33–1.40)
**Disability**		
No	1.00	
Yes	0.82	(0.70–0.97)
**Charlson comorbidity index**		
0	1.00	
1	1.24	(1.11–1.40)
≥ 2	1.53	(1.34–1.75)
**Cancer type**		
Stomach	1.00	
Liver	1.16	(0.93–1.45)
Colorectal	1.34	(1.07–1.66)
Lung or bronchus	1.07	(0.83–1.38)
The other	1.40	(1.18–1.66)
**Survival years**		
5	1.00	
6–7	5.18	(2.00–13.41)
8–9	10.27	(3.89–27.07)
≥ 10	21.02	(8.06–54.81)
**5th survival year after cancer diagnosis**		
2009	1.00	
2010	1.11	(0.94–1.32)
2011	1.15	(0.97–1.38)
2012	1.00	(0.83–1.20)
2013	1.16	(0.96–1.41)
2014	1.04	(0.85–1.27)
2015	2.68	(1.92–3.74)
2016	2.21	(1.54–3.17)
2017	4.39	(2.77–6.96)
2018	3.38	(2.03–5.65)
2019	15.18	(5.60–41.17)

Table 3 shows the subgroup analysis of the dyslipidemia risk by independent variables. New-onset dyslipidemia tended to be at greater risk in women, participants aged ≥ 80 years, high-income class, participants with disability, patients with a higher CCI, and fifth-year cancer survivor (women, HR = 1.22, 95% CI = 1.07–1.40; people ≥ 80 years old, HR = 1.40, 95% CI = 0.96–2.04; high-income class, HR = 1.25, 95% CI = 1.08–1.45; those with a disability, HR = 1.50, 95% CI = 1.09–2.06; patients with CCI two or higher, HR = 1.38, 95% CI = 1.11–1.70; fifth-year cancer survivor, HR = 11.84, 95% CI = 2.73–51.37) than each counterpart (men, younger individuals, low-income class, those without disability, no or low CCI, and cancer survivors for six years or more).

**Table 3:** Cox proportional hazards regression analysis of the dyslipidemia risk by independent variables

**Variables**	**Dyslipidemia**		
	**Risk of new onset**		
	**Medically vulnerable areas**		
	**Non-vulnerable**	**Vulnerable**	
	**Adjusted HR**	**Adjusted HR**	**95% CI**
**Sex**			
Men	1.00	1.20	(1.03–1.39)
Women	1.00	1.22	(1.07–1.40)
**Age**			
20–39	1.00	0.95	(0.58–1.55)
40–59	1.00	1.16	(0.98–1.37)
60–79	1.00	1.22	(1.06–1.41)
≥ 80	1.00	1.40	(0.96–2.04)
**Household income**			
High	1.00	1.25	(1.08–1.45)
Mid	1.00	1.21	(1.01–1.45)
Low	1.00	1.20	(0.96–1.50)
**Disability**			
No	1.00	1.19	(1.07–1.32)
Yes	1.00	1.50	(1.09–2.06)
**Charlson comorbidity index**			
0	1.00	1.13	(0.98–1.31)
1	1.00	1.30	(1.09–1.55)
≥ 2	1.00	1.38	(1.11–1.70)
**Survival years**			
5	1.00	11.84	(2.73–51.37)
6–7	1.00	1.23	(0.82–1.84)
8–9	1.00	1.03	(0.78–1.37)
≥ 10	1.00	1.17	(1.05–1.31)

**Fig. 2:**
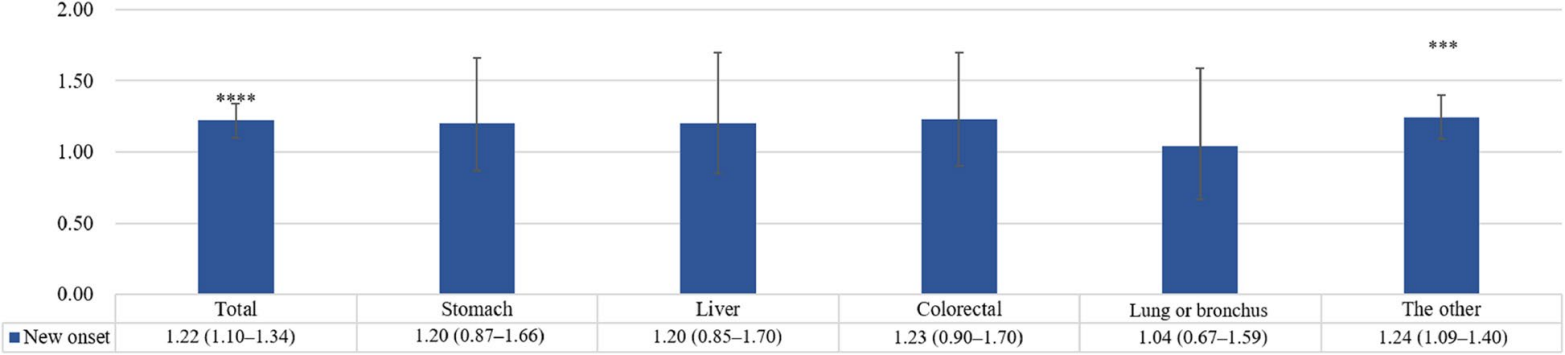
Cox proportional hazards regression analysis of the dyslipidemia risk by cancer types in medically vulnerable areas. Reference group: Healthcare non-vulnerable areas. *: *p* ≤ 0.05, **: *p* ≤ 0.01, ***: *p* ≤ 0.001, ****: *p* ≤ 0.0001

Figure 2 indicates the new-onset dyslipidemia according to cancer type in MUA. The risk of new-onset dyslipidemia in MUA compared with non-MUA was 1.20 times (95% CI = 0.87–1.66) higher in stomach cancer, 1.20 times (95% CI = 0.85–1.70) in liver cancer, 1.23 times (95% CI = 0.90–1.70) in colorectal cancer, 1.04 times (95% CI = 0.67–1.59) in lung or bronchus cancer, and 1.24 times (95% CI = 1.09–1.40) in other cancer.

## Discussion

This study demonstrated the impact of the occurrence of new-onset dyslipidemia in Korean adult cancer survivors for five years or more living in MUA. Cancer survivors for five years or more living from MUA have a higher risk of new-onset dyslipidemia than those from non-MUA. This higher risk was associated with women, old age, high income, disability, numerous or serious complications, and fifth-year cancer survivors. Colorectal cancer survivors of MUA tended to have a higher risk of dyslipidemia compared to other cancer types, although it was not prominent.

The annual incidence of new dyslipidemia per 1000 cancer survivors was 57.53 in MUA, 1.16 times higher than that in non-MUA. This health disparity may be attributed to chronic disease, exacerbated by cancer survivors from MUA because of poor access to healthcare. In Korea, medical institutions in rural areas account for 12.9% of those in urban areas [2]. The average number of beds per region was 1,480; however, the variation was extremely large, with a minimum of 8 and a maximum of 6,301.3 [31].

The risk of dyslipidemia in MUA was 1.22 times higher for new-onset than that in non-MUA. Dyslipidemia is treated with lifestyle changes and medication. MUA residents often use out-of-town medical care because of the disparities in medical quality. In Korea, 50.6% of medical institutions are biased toward metropolitan areas [32]. From 2010 to 2020, the proportion of non-metropolitan patients in tertiary general hospitals located in the metropolitan area increased by 3.2% among outpatients. There were 43 MUA without hospital-level regional emergency medical institutions and 93 MUA without emergency specialists [33]. In other words, dyslipidemia has the potential to lead to medical use in non-MUA or unmet medical care because of patient demand or insufficient medical resources in MUA.

Some factors were more prominent regarding the risk of dyslipidemia than their counterparts in MUA. Women tended to have a higher incidence of dyslipidemia from low-density lipoprotein cholesterol (LDL-C) [34]. Diabetes and excessive saturated fat intake are closely related to high LDL-C levels [35, 36]. In postmenopausal women, hormonal changes lead to much higher LDL-C serum levels [37]. Additionally, the percentage of women with excessive energy intake increased 1.88-fold over 9 years in South Korea (2007: 10.0%, 2015: 18.8%) [37]. Overweight and obese adults were more prevalent in lower-income groups in cities, however conversely, there were many nutritional inequalities among higher-income groups in rural areas [38]. Further research is needed to determine whether there is a difference in the factors affecting intake by sex and region.

Physical predisposing factors such as aging, disability, and comorbidities are also susceptible for dyslipidemia in MUAs. Dyslipidemia is a chronic disease, and its continuous management is important. Nevertheless, rural areas, which account for most MUAs, have inconvenient transportation to facilities, with physical distances to medical institutions being long and infrequent ships or buses. In addition, regardless of age or health status, the livelihoods of most residents in MUA are agriculture and fishing, which are physically demanding. As the physical labor group usually experiences considerable physical fatigue, the perception threshold of health deterioration is low, and, therefore, healthcare may be delayed. In addition, lack of exercise infrastructure, early bedtime, morning work, and minimal living areas can limit their exercise.

Colorectal cancer survivors tended to be at greater risk for dyslipidemia than survivors of other cancer types, although this was not statistically significant. There have been previous studies that directly or indirectly exist potential mechanisms for the relationship between colorectal cancer and dyslipidemia [8, 39]. Nevertheless, the association between colorectal cancer and dyslipidemia has not been confirmed, and the direction of association found in previous studies has changed over time [8, 40]. Dyslipidemia may be caused by the remaining eating habits of colorectal cancer survivors who had dietary problems, such as excessive intake of animal fat or saturated fat [41, 42]. However, dietary issues may not be sufficient to explain the etiology, and further studies are needed in the future.

This study had some limitations. First, the data were not adjusted for disease stage, duration, or medication regimen. Therefore, the frequency of medical use owing to dyslipidemia was not analyzed. Second, factors such as diet and genetics could not be identified because of a lack of data. Not all factors influencing the development of dyslipidemia were considered in this study.

## Conclusions

Cancer survivors for five years or more had a higher risk of new-onset dyslipidemia in MUA. Cancer survivors of the female sex and older age in MUA, with high household income, disability, numerous complications, and fifth-year cancer survivors were extremely vulnerable to new-onset dyslipidemia. Additionally, colorectal cancer survivors tended to have a dyslipidemia risk than survivors of other types of cancer. There is a need for health measures to prevent and alleviate dyslipidemia in cancer survivors residing in MUA. In addition, a person-centered approach should be adopted, considering the demographic and disease history of cancer survivors.

## Data Availability

The Korean National Health Insurance Service–National Sample Cohort is a public, open-access database. It is based on the health insurance claim data of all Koreans, and the sample cohort is available for public purposes and scientific research. The authors do not have permission to share these data. The sample cohort data are available after acceptance of approval for use by the National Health Insurance Service (https://nhiss.nhis.or.kr/bd/ab/bdaba000eng.do).

## Abbreviations

Cis: Confidence intervals
CCI: Charlson comorbidity index
HDL: High-density lipoprotein
HR: Hazard ratios
IRB: Institutional Review Board
ICD-10: International Classification of Diseases 10^th^
LDL: Low-density lipoprotein
MUA: Medically underserved areas
NHIS: National Health Insurance Service
OECD: Organization for Economic Cooperation and Development
PARC: Position value for the relative comparison
TG: Triglycerides
SD: Standard deviation
